# The role of vancomycin in addition with colistin and meropenem against colistin-sensitive multidrug resistant *Acinetobacter baumannii* causing severe infections in a Paediatric Intensive Care Unit

**DOI:** 10.1186/s12879-015-1133-3

**Published:** 2015-09-30

**Authors:** Giancarlo Ceccarelli, Alessandra Oliva, Gabriella d’Ettorre, Alessandra D’Abramo, Elena Caresta, Caterina Silvia Barbara, Maria Teresa Mascellino, Paola Papoff, Corrado Moretti, Vincenzo Vullo, Paolo Visca, Mario Venditti

**Affiliations:** Department of Public Health and Infectious Diseases, University of Rome “Sapienza”, Viale del Policlinico 155, Rome, Italy; Pediatric Intensive Care Unit, Department of Pediatric Sciences, University of Rome “Sapienza”, Viale del Policlinico 155, Rome, Italy; Azienda Policlinico Umberto I, Viale del Policlinico 155, Rome, Italy; Department of Science, Roma Tre University, Viale G. Marconi 446, Rome, Italy

**Keywords:** Pediatric Intensive Care Unit (PICU), *Acinetobacter baumannii*, Multidrug resistant gram negatives, Colistin, Vancomycin, Synergism

## Abstract

**Background:**

*Acinetobacter baumannii* has been associated with high morbidity and mortality rates, even in pediatric patients. Therapeutic options are limited, especially when the strain is multidrug resistant.

**Methods:**

Clinical and microbiological analyses of 4 cases of systemic infections caused by multi drug resistant *A. baumannii* treated with colistin/vancomycin combination at a Pediatric Intensive Care Unit were performed in order to explore the potential synergistic activity of colistin plus vancomycin. All the patients were treated with colistin, meropenem and vancomycin.

**Results:**

Four severe infections due to MDR *A. baumannii* were observed. All patients treated with colistin/vancomycin combination had a positive outcome with no infection relapses. Most importantly, no significant adverse events related to the simultaneous administration of COL plus VAN were observed. In our *in-vitro* experiments, the synergistic effect of the combination COL plus VAN showed an early bactericidal activity even at VAN concentration of 16 mg/L, which reflects the serum trough concentrations obtained in patients.

**Discussion:**

An antimicrobial strategy based on the activity of colistin plus vancomycin was *in-vitro* and *in-vivo* effective in life-threatening infections caused by multidrug-resistant *A. baumannii* in a Pediatric Intensive Care Unit*,* in the absence of adverse effects. Colistin plus vancomycin were highly synergic and bactericidal against carbapenem-resistant, colistin sensitive *A. baumannii* whereas the addition of meropenem did not enhance the *in-vitro* activity of colistin plus vancomycin.

**Conclusions:**

Our results confirm existing data on the potential synergistic activity of a therapeutic strategy including colistin plus vancomycin and provide important new clinical information for its potential use as a therapeutic option against MDR *A. baumannii* infections, especially in the pediatric population.

**Electronic supplementary material:**

The online version of this article (doi:10.1186/s12879-015-1133-3) contains supplementary material, which is available to authorized users.

## Background

*Acinetobacter baumannii* has been recognized as a leading nosocomial pathogen associated with significant increase of length of hospitalization and mortality rates, even in the pediatric critical care units (PICUs) [1–5]. Moreover, therapeutic options are severely limited by the emergence of strains resistant to most antibiotics including carbapenems [6].

A few epidemic lineages of *A. baumannii* have been considered to be responsible for the majority of the hospital outbreaks worldwide, primarily the three international clonal lineages (ICLs) designated as ICL-I, ICL-II and ICL-III [7]. Resistance to several antibiotics, in particular carbapenems, is the hallmark of the most successful epidemic lineages [8]. Of note, an increasing incidence of carbapenem resistance has recently been reported among multi-drug resistant (MDR) ICL-II *A. baumannii* from central Italy, with the emergence in 2009 of a major clone carrying the *bla*OXA-23-like determinant [9].

Nowadays, the treatment of MDR *A. baumannii* infections represents a real challenge. Colistin (COL)-based combinations, with or without the addition of high dose of carbapenems, have been considered the milestone of the treatment; however, in consideration of the growing rate of resistance to several antimicrobial classes, innovative approaches against this microorganism have been investigated. Among these un-orthodox combinations, COL plus glycopeptides resulted to be effective both *in-vitro* and in animal models [10–13], with the potential additional advantage of preventing the development of COL resistance in COL sensitive strains [14, 15].

However, in the literature there are limited and anecdotal data regarding the use of this regimen in the clinical practice, especially in the pediatric patients. In fact*,* previous evidences were collected only throughout retrospective analyses.

Based on the potential synergistic activity of COL plus VAN, we report the *in-vitro* and *in-vivo* effectiveness of an antimicrobial strategy consisting of COL plus VAN plus meropenem (MEM) in four cases of systemic infections caused by MDR *A. baumannii* in a PICU.

## Methods

Over a 2-months period, four cases of systemic infections due to MDR *A. baumannii* treated with the combination colistin plus vancomycin in patients hospitalized at PICU of “Sapienza” University of Rome were observed. For each patient, clinical and microbiological data were recorded.

Written informed consent for the inclusion in this work was obtained from the patients or their legal guardians; according to local regulations, no Ethical Committee authorization is required in these cases [16, 17].

Isolates from clinical samples including blood, tracheal aspirate and broncoalveolar lavage underwent identification and antimicrobial susceptibility testing using the VITEK-2 (Bio-Merieux, Marcy l’Etoile, France) automated system.

Molecular analyses were performed in two strains whereas all the microbiological analyses were performed only on the case index strain.

To define the *A. baumannii* sequence group (SG), two multiplex PCRs designed to selectively amplify group 1 or group 2 alleles of the *ompA*, *csuE* and *bla*OXA-51-like genes were performed, and allelic profiles were interpreted according to Turton et al. [18]. The presence of four groups of OXA-carbapenemase genes (namely *bla*OXA-23-like, *bla*OXA-24-like, *bla*OXA-51-like and *bla*OXA-58-like) was detected by PCR using a multiplex assay as previously described [19, 20].

Minimal inhibitory concentrations (MICs) and logarithmic minimal bactericidal concentrations (MBClog) of MEM, COL, VAN and rifampin (RIF) were determined by broth macrodilution method (BMD) in cation-adjusted Mueller Hinton broth (CAMHB) according to the Clinical Laboratory Standards Institute (CLSI) guidelines [21]. For all COL testing, a polysorbate-80 (Sigma-Aldrich) final concentration of 0.002 % was used [22]. The MIC was defined as the lowest concentration of antibiotic that completely inhibited visible growth whereas the MBClog was defined as the lowest antimicrobial concentration which killed ≥99.9 % of the initial bacterial count (i.e., ≥3 log10 CFU/ml) at 24 h.

Synergy tests were performed throughout the checkerboard method at different concentrations of the following antibiotic combinations: COL + MEM, COL + RIF, COL + VAN, MEM + VAN.

Complete synergism was defined as FIC-index (FICI) ≤0.5, partial synergism as FICI >0.5 < 1, additivity as FICI ≥1 < 2, antagonism as FICI ≥2. A final inoculum of ~5 × 105 CFU/ml was used for all *in-vitro* experiments. In addition, the Susceptible Breakpoint Indices (SBPI) of the tested combinations was calculated [10]. To determine VAN and RIF susceptibility, the authors used breakpoints consistent with those set for Gram-positive organisms by the CLSI and EUCAST [10].

Furthermore, the activity of COL, VAN and MEM, alone and in combination, was investigated by time-kill studies using an initial inoculum of ~5 × 105 CFU/mL. As stated before, the time kill studies were conducted only on case index strain. At 2, 4, 6, 8 and 24 h time points, the number of CFU was counted. The following concentrations were used for killing tests: 1 × MIC COL, 1 × MIC VAN, 1 × MIC MEM, 0.5 × MIC COL + 0.25 × MIC VAN, 0.5 × MIC COL + 0.125 × MIC VAN, 0.5 × MIC COL + 0.5 × MIC MEM, 0.5 × MIC COL + 0.25 × MIC MEM, 0.5 × MIC COL + 0.25 × MIC VAN + 0.5 × MIC MEM and 0.5 × MIC COL + 0.125 × MIC VAN + 0.5 × MIC MEM.

Bactericidal activity was defined as a ≥3-log10 CFU/mL reduction of the initial bacterial count at each time point. Synergy was defined as a ≥2-log10 decrease in CFU/mL between the combinations and its most active constituent after 24 h. The detection limit was 10 CFU/mL.

## Results

### Case series

**Fig. 1:**
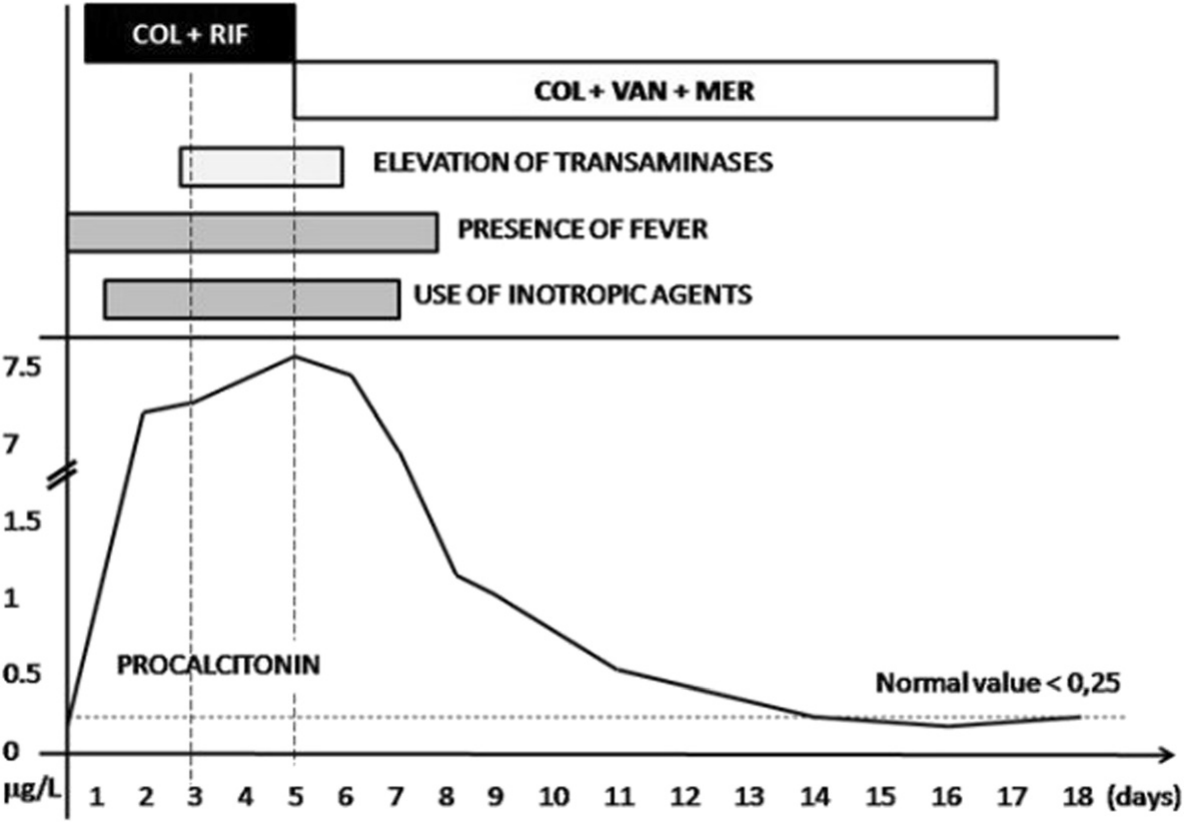
Clinical and therapeutic features of case-index MDR *A. baumannii* bacteremia. Procalcitonin levels increased up to 7 mg/L under treatment with COL + RIF, which was expression of treatment failure. Rather, it started to decrease under COL + VAN + MEM therapy and reached the normal value after 15 days of such therapy. The decreasing levels of procalcitonin following therapy with COL + VAN + MEM was expression of treatment success. COL: Colistin, VAN: Vancomycin, RIF: Rifampin, MEM: Meropenem

Four severe infections [2 bloodstream infections (BSIs), 1 ventilator associated pneumonia (VAP), 1 BSI plus VAP] due to MDR *A. baumannii* were observed at the PICU. Clinical and microbiological data of the index case are reported in Table 1. The index case was a newborn with a severe sepsis caused by MDR and COL sensitive *A. baumannii.* The patient was initially treated for 5 days with intravenous (i.v.) COL (6 mg/kg equivalent to 75,000 IU/kg per day, in three divided doses following a loading dose of 6 mg/kg) and RIF (10 mg/kg per day). However, due to worsening of the clinical conditions, VAN (40 mg/kg per day, in three divided doses) and MEM (60 mg/kg per day, in three divided doses) were added to COL, whereas RIF was stopped because of hepatic toxicity. A prompt clinical response was observed and the patient completed a 17 days treatment course. Subsequently, the patient developed a late onset uncomplicated urinary tract infection caused by the same MDR *A. baumannii* (Fig. 1 and Table 1).

**Table 1:** Clinical characteristics and microbiological data of patients

Patient	Age	Underlying disease	Diagnosis at admission	Type of infection	Treatment (duration, days)		Outcome^d^
					Initial	Definitive	
Case#1	3 mo^a^	Pierre Robin Syndrome	Septic shock (*A. baumannii* BSI)	BSI	RIF + COL (5)	VAN + COL + MEM (12)	Late relapse (UTI) Cured
Case#2	21 ys	Spastic tetraparesis; dysphagia	Respiratory failure^b^	VAP & BSI	RIF + COL (5)	VAN + COL + MEM (15)	Cured
Case#3	25 ys	Spastic tetraparesis	Respiratory failure^b^	BSI	VAN + COL + MEM (16)	VAN + COL + MEM^c^	Cured
Case#4	6 mo	None	ARDS	VAP	VAN + COL + MEM (19)	VAN + COL + MEM^c^	Cured

*COL* colistin, *VAN* vancomycin, *RIF* rifampin, *MEM* meropenem, *UTI* urinary Tract Infection, *BSI* bloodstream infection, *VAP* Ventilator-Acquired Pneumonia, *ARDS* Acute Respiratory Distress Syndrome

(^a^): Case-index; (^b^): caused by the underlying disease; (^c^): in cases #3-4 the initial therapy was the definitive one; (^d^): at least 3-months follow-up

Based on the favourable outcome of the index case, the 3 following cases were treated with the combination COL plus VAN plus MEM. In particular, in case#2 this strategy was started after an initial failure of the COL plus RIF treatment whereas the remaining two cases were both treated with the triple combination as initial therapy. The patients had a positive outcome with no infection relapses. Most importantly, no significant adverse events related to the simultaneous administration of COL plus VAN were observed.

### Microbiological studies

Molecular analyses were performed on two clinical strains (case index and case#2). Both isolates were PCR-positive for the *bla*oxa-51-like gene, confirming their identity as *A. baumannii* [23].

Multiplex PCRs for identification of the SG yielded the 111 allelic profiles (corresponding to SG 1 according to Turton et al. [18]), indicating that both isolates belonged to the ICL-II (Additional file 1: Figure S1).

Carbapenem resistance was associated with the presence of the *bla*OXA-23-like carbapenem hydrolyzing oxacillinase gene (Additional file 2: Figure S2).

According to the VITEK-2 system, the strains of the four patients were similar, and were resistant to gentamicin, ciprofloxacin, amoxicillin/clavulanate, cefotaxime, ertapenem, impenem, meropenem, trimethoprim/sulfamethoxazole, tigecycline and sensitive only to COL (MIC = 0.5 mg/L).

Additional microbiological assays to assess the potential synergism of COL plus VAN were conducted only on the index strain.

Using the MBD method, MICs/MBCs were 1/1, 128/256, 8/128 and 128/256 mg/L for COL, MEM, RIF and VAN, respectively. A complete synergism (FIC index <0.5) was observed for COL + VAN, COL + MEM and COL + RIF whereas the combination MEM + VAN showed additivity. The SBPI values were 8.12, 16.06, 12 and 0.04 for COL + VAN, COL + MEM, COL + RIF and MEM + VAN, respectively.

In particular, the combination COL + VAN showed an absence of growth at the following concentrations: 0.5 × MIC COL (0.5 mg/L) + 0.25 × MIC VAN (32 mg/L), 0.5 × MIC COL (0.5 mg/L) + 0.125 × MIC VAN (16 mg/L), 0.25 × MIC COL (0.25 mg/L) + 0.25 × MIC VAN (32 mg/L), 0.25 × MICCOL (0.25 mg/L) + 0.125 × MIC VAN (16 mg/L).

The combination COL + MEM + VAN at the concentrations of 0.5 × MIC COL (0.5 mg/L) + 0.5 × MIC MEM (64 mg/L) + 0.25 × MIC VAN (32 mg/L) and 0.5 × MIC COL (0.5 mg/L) + 0.5 × MIC MEM (64 mg/L) + 0.125 × MIC VAN (16 mg/L) showed *in-vitro* activity. Given the high bacteriostatic activity of the combinations COL + VAN and COL + VAN + MEM, we decided to perform bactericidal analyses by using different concentration of such antimicrobials.

**Fig. 2:**
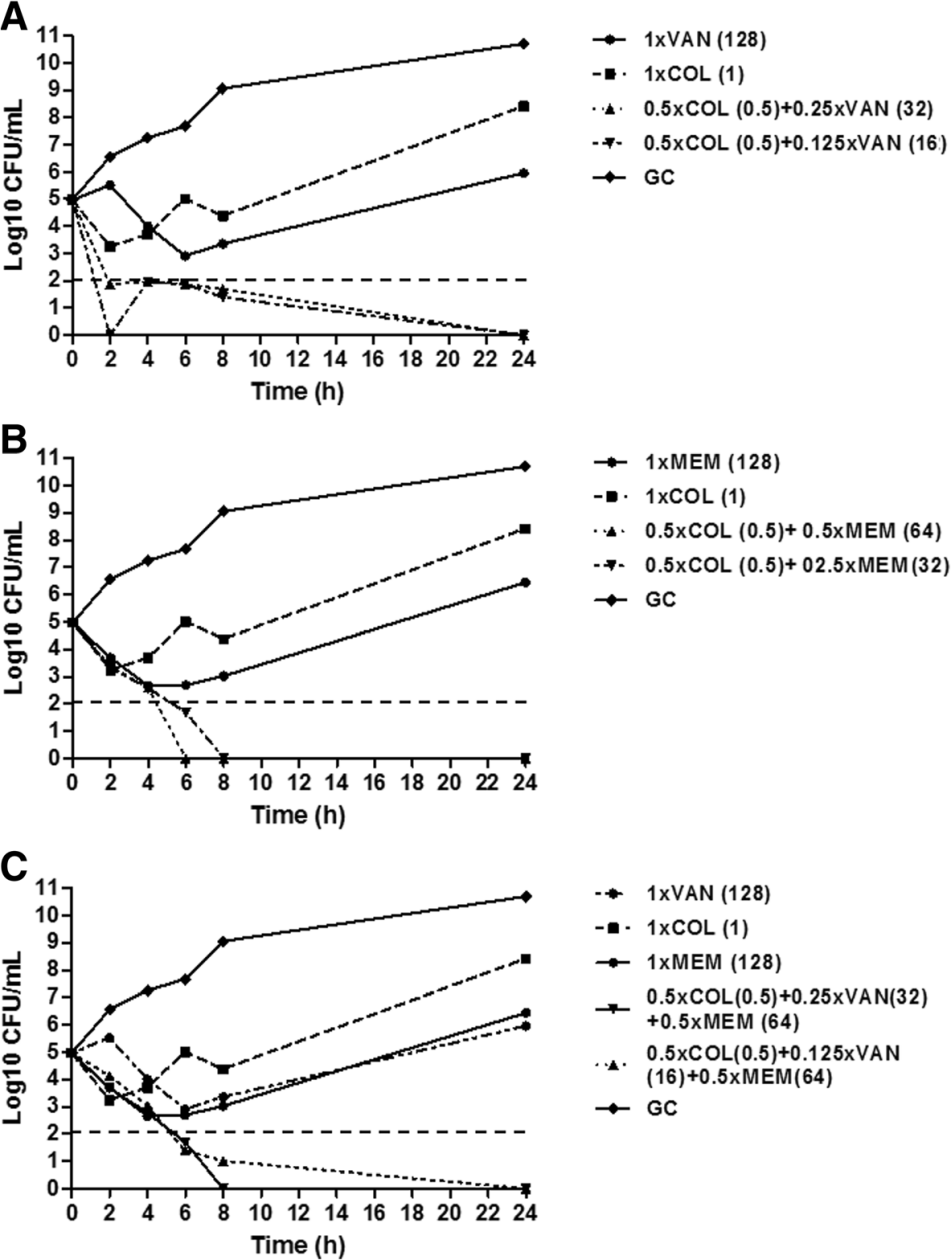
Time–kill studies for colistin (COL), meropenem (MEM) and vancomycin (VAN), alone and in combination, against MDR *A. baumannii.* **a** The bactericidal activity of COL + VAN is represented. Values in bracket represent the actual concentration (mg/L). **b** The bactericidal activity of COL + MEM is represented. Values in bracket represent the actual concentration (mg/L). **c** The bactericidal activity of COL + MEM + VAN is represented. The actual concentrations of the triple combinations are: 0.5 × MIC COL (0.5 mg/L) COL + 0.25 × MIC VAN (32 mg/L) + 0.5 × MIC MEM (64 mg/L) and 0.5 × MIC COL (0.5 mg/L) COL + 0.125 × MIC VAN (16 mg/L) + 0.5 × MIC MEM (64 mg/L). The dashed horizontal line represents a reduction of 3 log10 cfu/mL compared with the initial bacterial count. GC, growth control

In the killing experiments, VAN, MEM and COL alone showed only a slight decrease of CFU/mL at 2 h, 4 h, and 6 h time points; however, a significant re-growth was observed at 24 h for all antimicrobials (Fig. 2).

When the combination COL + VAN was tested, bactericidal activity was observed after 2 h at concentrations of 0.5 × MIC COL (0.5 mg/L) + 0.25 × MIC VAN (32 mg/L) and 0.5 × MIC COL (0.5 mg/L) + 0.125 × MIC VAN (16 mg/L), which was maintained until 24 h (Fig. 2a). In addition, these combinations were synergic at 24 h. Of note, the latter combination reflects the serum trough concentrations of VAN which can be achieved during therapy (16 mg/L).

The combinations 0.5 × MIC COL (0.5 mg/L) + 0.5 × MIC MEM (64 mg/L) and 0.5 × MIC COL (0.5 mg/L) + 0.25 × MIC MEM (32 mg/L) showed a bactericidal activity at 6 h, which was observed until 24 h (Fig. 2b).

The triple combinations 0.5 × MIC COL (0.5 mg/L) COL + 0.5 × MIC MEM (64 mg/L) + 0.25 × MIC VAN (32 mg/L) and 0.5 × MIC COL (0.5 mg/L) COL + 0.5 × MIC MEM (64 mg/L) + 0.125 × MIC VAN (16 mg/L) were found to be bactericidal at 6 h, with absence of growth at 24 h (Fig. 2c).

## Discussion

In this report, we described the *in-vitro* and *in-vivo* effectiveness of a combination containing COL, MEM and VAN against MDR *A. baumannii* causing systemic infections at a PICU.

Nowadays, the ability of *A. baumannii* to rapidly acquire antibiotic resistance has been recognized as an important therapeutic challenge. Therefore, the use of COL has been recently reviewed and combinations of COL plus RIF or tigecycline or ampicillin-sulbactam have been used in critically ill adults and children. However, tigecycline safety and efficacy have not been established yet in pediatric patients, and its use in these subjects is so far not recommended [24–29].

The usefulness of a combination therapy has been highlighted by a recent clinical trial in patients with life-threatening infections due to MDR *A. baumannii* [30]*.* Despite the 30-day mortality was not reduced by the addition of RIF to COL (at a daily dosage of 6 MUI), an increased rate of *A. baumannii* eradication was observed relative to COL alone [30].

The *in-vitro* synergistic activity of COL-based regimens in COL-susceptible and COL-resistant *A. baumannii* strains is an area of active investigation [25–29]. Among innovative approaches, the combination of COL plus other antimicrobials usually used against Gram-positive bacteria has recently gained interest. Potent *in-vitro* synergistic interactions between COL and daptomycin have been described, although the beneficial effects of this combination were limited only to COL susceptible isolates [31]. A study on the activity of COL plus telavancin (a novel lipoglycopeptide antibiotic with a narrow anti-Gram-positive spectrum) reported a marked synergism both in checkerboard and time–kill assays against COL-susceptible MDR *A. baumannii* isolates [32].

However, COL + VAN is regarded to as the most promising combination. A pioneer study by Gordon et al. first described an unexpected potent synergism and a sustained bactericidal activity of the VAN + COL combination against MDR COL-sensitive *A. baumannii* [10], and similar effects were later reported for COL-resistant strains [12]. Interestingly, the bactericidal activity was only slightly delayed in COL-resistant compared to the susceptible isolates, suggesting that the mechanisms responsible for COL resistance in *A. baumannii* strains do not significantly affect the synergism of the VAN + COL combination. However, few anecdotal data on the use of this regimen in pediatric patients are available, and these were collected only through retrospective analyses [33, 34].

The rationale of using COL plus VAN resides on the hypothesis that COL increases the permeability of the outer membrane, thereby enhancing the antibacterial activity of large sized hydrophobic molecules, such as VAN, which are normally excluded by the Gram-negative outer membrane. Thus, the membrane-perturbing properties of COL could allow VAN to reach its periplasmic target at inhibitory concentrations [10]. The importance of the cell-permeabilizing properties of COL in determining synergisms with unconventional antimicrobials is consistent with the absence of *in-vitro* synergism observed in our study for the MEM + VAN combination.

Notably, we observed a clear clinical and microbiological response in all the patients treated with the new therapeutic approach consisting of COL plus VAN plus MEM, in the absence of adverse events. Our decision to combine VAN with COL and MEM was motivated by the lack of response to the COL plus RIF combination in the index case. This led us to suppose that a new infection was simultaneously present, and pending microbiological data, broad spectrum empiric therapy was started. Considering that no microorganisms other than MDR *A. baumannii* were documented, we were impressed by the quick clinical response of *A. baumannii* BSI to COL + VAN + MEM, and we wondered which role the glycopeptide and carbapenem could have had in this combination.

Clinical results were supported by *in-vitro* studies, showing not only a bacteriostatic synergistic activity of COL plus VAN, but also a potent bactericidal activity. These findings corroborate existing data on the synergistic activity of COL plus VAN [33, 34], thus providing novel clinical information for its potential use as a viable therapeutic option against MDR *A. baumannii* infections in the pediatric population.

Nevertheless, it should be pointed out that all the patients had also received MEM together with COL and VAN. Thus, the contribution of MEM to the clinical resolution of the infections should be taken into account. Time-kill results showed that the addition of MEM to COL + VAN did not enhance the bactericidal activity of the combination COL + VAN.

Interestingly, the combination of COL + VAN resulted to be more rapidly bactericidal than both COL + MEM and COL + VAN + MEM combinations. However, the combination COL + MEM showed an absence of growth at 6 h, whereas COL + VAN, although quickly bactericidal (i.e., at 2 h), still showed some bacterial growth at 6 h and 8 h. The meaning of these slight differences between COL + VAN and COL + MEM observed *in vitro*, and their possible clinical implications, deserve further investigations.

In our *in-vitro* experiments, the synergistic effect of the combination COL plus VAN was impressive, with an early bactericidal activity even at VAN concentration of 16 mg/L, which reflects the serum trough concentrations obtained in patients [35]. Moreover, the addition of high concentrations of MEM, which are not easily achieved in patient’s serum despite prolonged infusion, did not enhance the *in-vitro* synergistic activity of COL + VAN. These results might be of crucial importance in the clinical practice, considering the deleterious effects that extensive use carbapenems has in the critical care setting, such as the selection of carbapenemase producing *K. pneumoniae*, carbapenem-resistant *Pseudomonas aeruginosa* and *Stenotrophomonas maltophilia*. In our opinion, the implementation of a therapeutic strategy aimed at treating infections caused by MDR *A. baumannii* based on the combination COL + VAN, without the addition of MEM, should be investigated in larger studies, in order to confirm these intriguing results in terms of clinical and microbiological effectiveness.

Although molecular analyses were performed only on two isolates, the temporal and spatial spread of infections into the PICU is suggestive of an outbreak caused by a single clone of MDR *A. baumannii*. In fact, both isolates belonged to the ICL-II, carried the *bla*OXA-23-likecarbapenemase gene and showed the MDR phenotype. Moreover, they shared the typical features of the main epidemic ICL-II clone widespread in central Italy [9, 36]. It is therefore tempting to speculate that the therapeutic protocol reported in this study would prove to be successful for the treatment of other infections caused by this worrisome *A. baumannii* clone, not only in the PICU setting.

Whether the COL + VAN combination could be associated with an increased risk of renal toxicity is still matter of discussion. In fact, one study reported that the rate of acute kidney injury is significantly higher in the group treated with COL plus VAN than in those receiving only COL alone [33], whereas another study reported similar nephrotoxicity in patients treated with and without a COL-glycopeptide combination [34]. In the literature, there are limited data regarding the potential nephrotoxicity of COL-based combinations in pediatric patients. However, it has been shown that only a minority of critically ill children receiving intravenous COL for MDR Gram negative infections developed renal toxicity, even when COL was co-administered with VAN [37].

## Conclusion

Our study provides additional evidence that the innovative combination of COL plus VAN, together with the possible association of MEM, could represent a life-saving therapy in selected cases of severe infections caused by MDR *A. baumannii.* Although our data refer to only four cases of life-threatening infections likely due to a single clone of MDR *A. baumannii*, it is plausible that this strategy will serve as an effective and safe therapeutic option in severe infections caused by MDR *A. baumannii*.

### Ethical standards

Written informed consent to the inclusion in this work was obtained from the patients or their legal guardians.

## Additional files

Additional file 1: Figure S1. Multiplex PCR analysis of *ompA, csuE* and *bla*OXA-51-like alleles of the two *A. baumannii* isolates. MP1 and MP2 indicate multiplex PCRs for group 1 and 2 alleles, respectively (Turton et al. [18]). The presence of amplicons of 355 bp (*ompA*), 559 bp (*bla*OXA-51-like) and 702 bp (*csuE*) are typical of SG 1, corresponding to the ICL-II. M, molecular weight marker (size in base pairs). Patient 1 is the index case. (JPEG 51 kb) Additional file 2: Figure S2. PCR-based detection of carbapenemase genes. For both *A. baumannii* isolates, the predicted amplicons of 353 bp and 501 bp were obtained for *bla*OXA51-like and *bla*OXA-23-like genes, respectively. M, molecular weight marker (size in base pairs). Patient 1 is the index case. (JPEG 56 kb)

## Abbreviations

BMD: Broth macrodilution method
BSI: Blood-stream infections
CAMHB: Cation-adjusted Mueller Hinton broth
CLSI: Clinical Laboratory Standards Institute
COL: Colistin
i.v.: Intravenous
MBClog: Logarithmic minimal bactericidal concentrations
MBL: Metallo-betalactamase
MDR: Multidrug-resistant
MEM: Meropenem
MICs: Minimal inhibitory concentrations
OXA: Oxacillinases
PICU: Pediatric intensive care unit
RIF: Rifampin
VAN: Vancomycin
VAP: Ventilator associated pneumonia
