# Supplementary figures and images for: The role of vancomycin in addition with colistin and meropenem against colistin-sensitive multidrug resistant Acinetobacter baumannii causing severe infections in a Paediatric Intensive Care Unit
Source: BMC Infect Dis. 2015 Sep 30;15:393. doi: 10.1186/s12879-015-1133-3 (PMC4589198; doi:10.1186/s12879-015-1133-3)

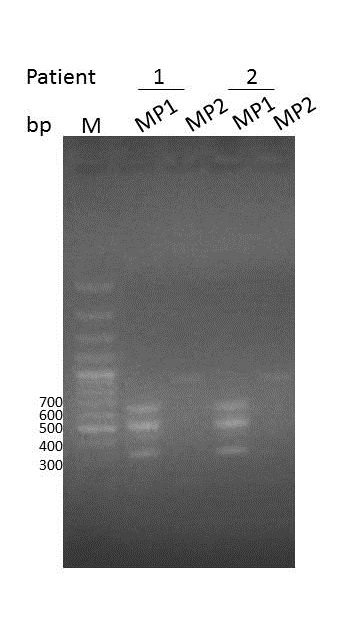

Supplement: Additional file 1: Figure S1. — Multiplex PCR analysis of ompA, csuE and blaOXA-51-like alleles of the two A. baumannii isolates. MP1 and MP2 indicate multiplex PCRs for group 1 and 2 alleles, respectively (Turton et al. [18]). The presence of amplicons of 355 bp (ompA), 559 bp (blaOXA-51-like) and 702 bp (csuE) are typical of SG 1, corresponding to the ICL-II. M, molecular weight marker (size in base pairs). Patient 1 is the index case. (JPEG 51 kb) [file 12879_2015_1133_MOESM1_ESM.jpeg]

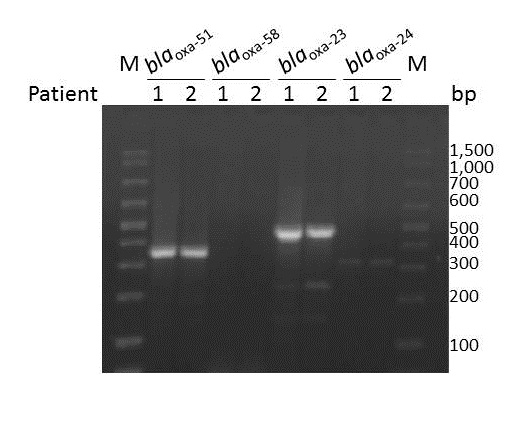

Supplement: Additional file 2: Figure S2. — PCR-based detection of carbapenemase genes. For both A. baumannii isolates, the predicted amplicons of 353 bp and 501 bp were obtained for blaOXA51-like and blaOXA-23-like genes, respectively. M, molecular weight marker (size in base pairs). Patient 1 is the index case. (JPEG 56 kb) [file 12879_2015_1133_MOESM2_ESM.jpeg]
